# Causes of death in men with localized prostate cancer: a nationwide, population‐based study

**DOI:** 10.1111/bju.13059

**Published:** 2015-05-15

**Authors:** Mieke Van Hemelrijck, Yasin Folkvaljon, Jan Adolfsson, Olof Akre, Lars Holmberg, Hans Garmo, Pär Stattin

**Affiliations:** ^1^ Division of Cancer StudiesCancer Epidemiology GroupSchool of MedicineKing's College LondonLondonUK; ^2^ Regional Cancer CentreUppsala ÖrebroUppsalaSweden; ^3^ CLINTEC DepartmentKarolinska InstitutetStockholmSweden; ^4^ Clinical Epidemiology UnitDepartment of Medicine (Solna)Karolinska InstituteStockholmSweden; ^5^ Department of Surgical SciencesUppsala UniversityUppsalaSweden; ^6^ Department of Surgical and Perioperative Sciences, Urology and AndrologyUmeå UniversityUmeåSweden

**Keywords:** comorbidities, prostate cancer death, curative treatment, localized disease

## Abstract

**Objective:**

To detail the distribution of causes of death from localized prostate cancer (PCa).

**Patients and Methods:**

The database PCBase Sweden links the Swedish National Prostate Cancer Register with other nationwide population‐based healthcare registers. We selected all 57 187 men diagnosed with localized PCa between 1997 and 2009 and their 114 374 PCa‐free control subjects, matched according to age and county of residence. Mortality was calculated using competing risk regression analyses, taking into account PCa risk category, age and Charlson comorbidity index (CCI).

**Results:**

In men with low‐risk PCa, all‐cause mortality was lower compared with that in corresponding PCa‐free men: 10‐year all‐cause mortality was 18% for men diagnosed at age 70 years, with a CCI score of 0, and 21% among corresponding control subjects. Of these cases, 31% died from cardiovascular disease (CVD) compared with 37% of the corresponding control subjects. For men with low‐risk PCa, 10‐year PCa‐mortality was 0.4, 1 and 3% when diagnosed at age 50, 60 and 70 years, respectively. PCa was the third most common cause of death (18%), after CVD (31%) and other cancers (30%). By contrast, PCa was the most common cause of death in men with intermediate‐ and high‐risk localized PCa.

**Conclusions:**

Men with low‐risk PCa had lower all‐cause mortality than PCa‐free men because of lower CVD mortality, driven by early detection selection; however, for men with intermediate‐ or high‐risk disease, the rate of PCa death was substantial, irrespective of CCI score, and this was even more pronounced for those diagnosed at age 50 or 60 years.

## Introduction

The advent of PSA testing has led to a rapid rise in the incidence of low‐risk and intermediate‐risk prostate cancer (PCa) in Sweden and elsewhere 1, 2. Men with low‐risk PCa have a low risk of death from PCa for up to 15 years after the date of diagnosis 3, 4. In the National Prostate Cancer Register (NPCR) of Sweden, men with conservatively treated low‐risk PCa (clinical local stage T1–2, Gleason score 2–6, PSA <10 ng/mL) had a 9% risk of PCa death and a 50% risk of death from other causes after 15 years of follow‐up 5. Despite the 9% PCa mortality, all‐cause mortality for men with low‐risk PCa, who were otherwise healthy, as indicated by a Charlson comorbidity index (CCI) score of 0, was identical to that of matched PCa‐free men. To further investigate causes of death among men with localized PCa and PCa‐free men, we used data in PCBaSe Sweden that originated from nationwide, population‐based healthcare registers and demographic databases for 57 187 men with localized PCa diagnosed between 1997 and 2009 and 114 374 matched PCa‐free men.

## Patients and Methods

### Study Population and Data Collection

In 2010, the NPCR of Sweden was linked to a number of other population‐based registers via the use of the Swedish personal identity number 6. The resulting database, PCBaSe Sweden 2.0, also includes a control series of men free of PCa at the time of sampling. The controls were randomly selected from men who matched an index case by county of residence and birth year 6. We included men who were diagnosed with localized PCa between 1997 and 2009, who had not received androgen deprivation therapy as primary treatment (*n* = 57 187), and their matched controls (*n* = 114 374). Men on androgen deprivation therapy (*n* = 17 537) were excluded because we had no additional information available on why these men with localized PCa were being treated with this therapy. Our focus was on men with localized disease treated with the standard treatment options available: surveillance, radical prostatectomy or radiotherapy. PCa‐free men who were diagnosed with PCa during the follow‐up were kept in the control group as we aimed to compare risk and cause of death for men with similar baseline characteristics to a background population in which PCa risk was not excluded. Follow‐up was available until 31 December 2011.

The main outcome of interest was death as registered in the National Cause of Death Register 7. We specifically studied death from PCa (International Classification of Diseases [ICD]‐10: C61), other cancers (ICD‐10: C00‐99, apart from C61), cardiovascular disease (CVD; ICD‐10: I00‐I99), chronic obstructive pulmonary disease (ICD‐10: J40‐44), and collapsed other causes into one category. We included information on the following potential confounders: age at diagnosis, PCa risk category, delivered or planned primary treatment <6 months after date of diagnosis, household, level of education and comorbidity. The variable ‘household’ reflects a man's partnership status and was defined as not single, single with children, or single with no children. From the NPCR, which started in 1996 and captures 98% of all newly diagnosed, biopsy‐confirmed PCa cases as compared with the Swedish Cancer Registry 8, we had detailed information about tumour characteristics and primary treatment 8, 9. Prostate cancer risk categories were defined according to a modification of the National Comprehensive Cancer Network Guidelines 6, 10. More specifically, low‐risk localized PCa was defined as T1–2, Gleason score 2–6, and PSA <10 ng/mL, whereas intermediate risk localized PCa was defined as T1–2, Gleason score 7 and/or 10 < PSA <20 ng/mL, and high‐risk localized PCa was defined as T3 and/or Gleason score 8–10 and/or 20 < PSA <50 ng/mL. Information on household and level of education was taken from the Longitudinal Integration Database for Health Insurance and Labour Market Studies (or LISA by its Swedish acronym), a database that integrates existing data from the labour market and educational and social sectors 11. The CCI was calculated to assess the burden of concomitant disease and we used 17 groups of diseases with a specific weight (1, 2, 3 and 6) assigned to each disease category based on discharge diagnoses in the National Patient Register 12, 13. The sum of these weights resulted in three levels of CCI score: 0 for no comorbidity, 1 for mild, and 2+ for severe comorbidity 14.

### Statistical Analysis

Because PCa risk category at time of diagnosis is associated with socio‐economic factors and comorbidity 15, we first performed univariate and multivariate conditional logistic regression to analyse how education, household and CCI were associated with low‐, intermediate‐ and high‐risk localized PCa, respectively. Fine and Gray competing risk regression analyses were then used to estimate 10‐year cause‐specific mortality 17. We first ran a multivariate Cox proportional hazards model for death from all causes. All variables were statistically significantly associated with outcome: age, CCI, education level, household and PCa risk category (results not shown). Year of PCa diagnosis did not have a strong effect on death from all causes, and, after inclusion in the models specified below, the results did not alter. Year of diagnosis was therefore not taken into account in our predictive models. We decided to predict mortality by age category (50, 60 and 70 years), CCI (0 and 2+), and PCa risk category (PCa‐free control subjects and men with low‐, intermediate‐ and high‐risk localized PCa). Furthermore, to account for the strong association between age and death, all models included age as a second‐degree polynomial. To facilitate the interpretation of the results we kept education level and household constant at their intermediate category for all prediction models: intermediate education level (10–12 years of school) and men who were married or in a civil partnership. A distinction between single men with and without children was made because fatherhood and civil status affect healthcare‐seeking behaviour 17. Thus, the different prediction parameters used in our models were based on age (50, 60 and 70 years at time of diagnosis), household (married or in civil partnership), education level (10–12 years of school), and CCI (0 and 2+). Household and education level were kept constant, whereas results were shown for different levels of age, CCI and PCa risk categories. As mortality may be different for curatively treated men compared with all men diagnosed with localized PCa as a result of the effect of treatment, but also as a result of selection of otherwise healthy men for curative treatments, competing risk regression analyses were also performed for this subgroup.

All data management was performed using sas release 9.2 (SAS Institute, Cary, NC, USA) and all statistical analysis were performed with R version 2.15.1 (R Foundation for Statistical Computing, Vienna, Austria). The Research Ethics Board at Umeå University approved the project.

## Results

Of the 57 187 men diagnosed with localized PCa between 1997 and 2009, 23 460 (41%) had low‐risk disease, 20 124 (35%) intermediate‐risk and 13 603 (24%) high‐risk disease. The baseline characteristics of these men and their 114 374 PCa‐free matched control subjects are shown in Table 1. The proportion of PCa deaths in the PCa‐free control group was ~4.5%, which reflects the lifetime risk of PCa and known incidence:death rate of 3:1 to 4:1. The proportion of PCa deaths was not much higher compared with the PCa group in this study as all men had localized PCa.

**Table 1:** Baseline characteristics for men with localized prostate cancer (PCa) and matched PCa‐free control subjects

	Low‐risk PCa (*n* = 23 460)	Intermediate‐risk PCa (*n* = 20 124)	High‐risk PCa (*n* = 13 603)	No PCa (*n* = 114 374)
**Mean (** **sd** **) follow‐up time, years**	6.4 (3.2)	6.2 (3.3)	6.3 (3.4)	6.3 (3.3)
Age group				
<65 years	10 741 (45.8)	6 682 (33.2)	3 369 (24.8)	41 584 (36.4)
65–74 years	9 578 (40.8)	9 109 (45.3)	5 817 (42.8)	49 008 (42.8)
75–84 years	2 876 (12.3)	3 858 (19.2)	3 659 (26.9)	20 786 (18.2)
≥85 years	265 (1.1)	475 (2.4)	758 (5.6)	2 996 (2.6)
**Time period, ** ***n*** **(%)**				
1997–2000	3 445 (14.7)	3 658 (18.2)	3 706 (27.2)	209 (4.9)
2001–2003	5 015 (21.4)	4 175 (20.7)	3 051 (22.4)	690 (16.2)
2004–2006	7 479 (31.9)	5 682 (28.2)	3 444 (25.3)	1 483 (34.8)
2007–2009	7 521 (32.1)	6 609 (32.8)	3 402 (25.0)	1 877 (44.1)
**Treatment, ** ***n*** **(%)**				
No PCa				
Surveillance	10 907 (46.5)	7 726 (38.4)	5 753 (42.3)	114 374 (100.0)
Radical prostatectomy	9 515 (40.6)	8 218 (40.8)	2 695 (19.8)	
Radiotherapy	3 038 (12.9)	4 180 (20.8)	5 155 (37.9)	
**Household, ** ***n*** **(%)**				
Not single	17 638 (75.2)	14 753 (73.3)	9 710 (71.4)	77 494 (67.8)
Single with children	4 079 (17.4)	3 767 (18.7)	2 655 (19.5)	23 455 (20.5)
Single, no children	1 743 (7.4)	1 604 (8.0)	1 238 (9.1)	13 425 (11.7)
**Education, ** ***n*** **(%)**				
Low: <10 years	7 984 (34.0)	7 632 (37.9)	5 871 (43.2)	48 540 (42.4)
Intermediate: 10–12 years	9 248 (39.4)	7 613 (37.8)	4 792 (35.2)	41 024 (35.9)
High: >12 years	6 077 (25.9)	4 699 (23.4)	2 713 (19.9)	22 643 (19.8)
Missing	151 (0.6)	180 (0.9)	227 (1.7)	2 167 (1.9)
**CCI score, ** ***n*** **(%)**				
0	17 572 (74.9)	14 541 (72.3)	9 192 (67.6)	79 592 (69.6)
1	3 448 (14.7)	3 212 (16.0)	2 381 (17.5)	18 597 (16.3)
2+	2 440 (10.4)	2 371 (11.8)	2 030 (14.9)	16 185 (14.2)
**Death**				
PCa‐related	866 (3.7)	925 (4.6)	718 (5.3)	5 164 (4.5)
From other causes	2 314 (9.9)	3 307 (16.4)	4 033 (29.6)	19 260 (16.8)

PCa, prostate cancer; CCI, Charlson comorbidity index. All men were diagnosed/selected between 1997 and 2009 in PCBaSe Sweden 2.0.

Table 2 shows the risk of PCa according to educational level, household and comorbidity. Men who were married or in partnership and men with a high educational level had a higher risk of PCa in all three risk categories and men with no comorbidity had an elevated risk of intermediate‐risk PCa and a non‐statistically significant elevated risk of low‐ and high‐risk PCa.

**Table 2:** Univariate and multivariate odds ratios and 95% CIs for risk of low‐, intermediate‐, or high‐risk localized prostate cancer

	Univariate model		Multivariate model	
	OR	95% CI	OR	95% CI
**Low‐risk prostate cancer**				
***Education***				
High	1.00	Ref.	1.00	Ref.
Intermediate	0.90	0.87–0.93	0.91	0.88–0.94
Low	0.73	0.70–0.75	0.75	0.73–0.78
***Household***				
Not single	1.00	Ref.	1.00	Ref.
Single with children	0.83	0.80–0.86	0.84	0.82–0.87
Single, no children	0.63	0.60–0.66	0.65	0.62–0.69
***CCI***				
0	1.00	Ref.	1.00	Ref.
1	0.94	0.91–0.98	0.96	0.92–1.00
2+	0.85	0.82–0.89	0.97	0.92–1.02
**Intermediate‐risk prostate cancer**				
***Education***				
High	1.00	Ref.	1.00	Ref.
Intermediate	0.91	0.87–0.94	0.92	0.89–0.96
Low	0.75	0.72–0.78	0.78	0.75–0.81
***Household***				
Not single	1.00	Ref.	1.00	Ref.
Single with children	0.87	0.84–0.90	0.88	0.85–0.91
Single, no children	0.67	0.64–0.71	0.70	0.66–0.73
***CCI***				
0	1.00	Ref.	1.00	Ref.
1	0.94	0.90–0.97	0.95	0.91–0.99
2+	0.79	0.76–0.83	0.88	0.84–0.93
**High‐risk prostate cancer**				
***Education***				
High	1.00	Ref.	1.00	Ref.
Intermediate	0.91	0.87–0.96	0.92	0.88–0.97
Low	0.83	0.79–0.87	0.85	0.81–0.89
***Household***				
Not single	1.00	Ref.	1.00	Ref.
Single with children	0.87	0.84–0.91	0.88	0.85–0.92
Single, no children	0.76	0.72–0.81	0.78	0.74–0.83
***CCI***				
0	1.00	Ref.	1.00	Ref.
1	0.95	0.91–1.00	0.96	0.92–1.01
2+	0.87	0.83–0.91	0.95	0.90–1.01

OR, odds ratio; PCa, prostate cancer; CCI, Charlson comorbidity index.

**Figure 1:**
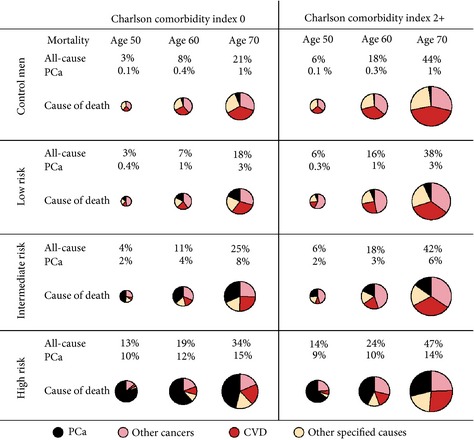
Predicted risk of death 10 years after prostate cancer (PCa) diagnosis for men aged 50, 60, and 70 years at date of diagnosis, by PCa risk category and Charlson comorbidity index (CCI). The risk of death from all causes is represented by the size of the pie charts. Each section of the pies represents the proportion of men who died from a specific cause estimated with Fine and Gray analyses.

Figure 1 shows the predicted 10‐year mortality after PCa diagnosis by categories of age, comorbidity, and PCa risk category, showing that mortality increased with each of these factors. For men with low‐risk PCa, all‐cause mortality was lower than for corresponding PCa‐free men. All‐cause mortality at 10 years was estimated to be 18% in men with low‐risk PCa diagnosed at age 70 years and with a CCI score of 0, and 21% among their controls. Of these cases, 31% died from CVD whereas 37% of their controls died from CVD (Fig. 1 and Table 3). The 10‐year PCa mortality rate was estimated to be 0.4% for men diagnosed at age 50 years with low‐risk PCa, 1% for those diagnosed at age 60 years, and 3% for those diagnosed at age 70 years. PCa was the third most common cause of death (18%), after CVD (31%) and other cancers (30%), in low‐risk PCa at 10 years after diagnosis (Table 3).

**Table 3:** Distribution of causes of death 10 years after prostate cancer (PCa) diagnosis for men aged 50, 60 and 70 years at date of diagnosis, by PCa risk category and Charlson comorbidity index score

Cause of death, %	CCI score 0			CCI score 2+		
	Age 50 years	Age 60 years	Age 70 years	Age 50 years	Age 60 years	Age 70 years
**Control subjects**						
PCa	2.2	4.1	5.8	0.8	1.5	2.1
Other cancers	37.8	38.3	30.1	36.2	36.3	28.6
CVD	22.6	28.8	36.6	27.8	35.1	43.4
Other specified causes	37.4	28.8	27.5	35.2	27.1	25.8
Overall mortality	2.7	8.5	21.0	5.8	18.0	44.0
**Low risk PCa**						
PCa	15.2	16.9	18.2	5.4	6.2	6.9
Other cancers	48.7	40.2	29.5	56.3	47.1	35.3
CVD	15.5	22.1	30.6	16.8	24.5	34.3
Other specified causes	20.6	20.8	21.6	21.5	22.2	23.5
Overall mortality	2.7	7.5	18.3	6.1	16.2	38.2
**Intermediate risk PCa**						
PCa	48.5	37.5	31.7	25.5	17.9	14.9
Other cancers	27.9	31.6	25.4	44.6	45.2	34.7
CVD	6.6	14.4	25.1	10.0	19.5	32.2
Other specified causes	17.0	16.5	17.8	19.8	17.4	18.2
Overall mortality	4.1	10.7	24.9	6.1	17.7	41.9
**High risk PCa**						
PCa	80.2	61.9	46.1	65.0	43.1	28.9
Other cancers	13.3	19.1	18.1	23.7	28.9	24.3
CVD	3.4	9.9	19.8	6.2	15.6	27.3
Other specified causes	3.2	9.1	16.0	5.1	12.5	19.5
Overall mortality	12.8	19.4	33.5	13.7	24.3	46.8

PCa, prostate cancer; CCI, Charlson comorbidity index; CVD, cardiovascular disease. The predictions were made based on median household status (married or in civil partnership) and intermediate educational level. Overall mortality for each category is also shown.

By contrast, men with intermediate‐ and high‐risk localized PCa had a higher all‐cause mortality than PCa‐free‐men. In men with high‐risk PCa,10‐year PCa mortality was estimated to be 10% among men diagnosed at age 50 years, 12% for those diagnosed at age 60 years, and 15% for those diagnosed at age 70 years. Prostate cancer was by far the most common cause of death: 80% of high‐risk men diagnosed at age 50 years who died, died from PCa and corresponding proportions were 62% for men diagnosed at age 60 years and 46% for those diagnosed at age 70 years (Table 3). A small proportion of control subjects were subsequently diagnosed with PCa during the follow‐up and PCa mortality was estimated to be 0.1% for men included as control subjects at age 50 years, 0.4% for men included at age 60 years and 1% at age 70 years (Fig. 1).

Similarly to all‐cause mortality, risk of PCa mortality increased with age and PCa risk category, regardless of comorbidity status. The 10‐year PCa mortality for men with intermediate‐risk disease was estimated to be very similar for men with a CCI score of 0 and a CCI score of 2+: 10‐year PCa mortality was 2, 4 and 8% at age 50, 60 and 70 years, respectively, for men with a CCI score of 0 and 2, 3 and 6% at age 50, 60 and 70 years, respectively, for men with a CCI score of 2+. In relative terms, the proportion of PCa death for men with a CCI score of 2+ was half of that of men with a CCI score of 0 (15 vs 32%; Table 3).

Data for chronic obstructive pulmonary disease are not shown in Fig. 1 and Table 3 as this was a very rare cause of death in all groups. The highest proportion (3%) of deaths from this disease was observed for PCa‐free men aged 70 years at baseline, with a CCI score of 2+. Consequently, death from chronic obstructive pulmonary disease was incorporated in the group including death from other causes.

Finally, we also estimated mortality for the subgroup of curatively treated men (Fig. S1). All‐cause mortality was slightly lower in these men compared with the total group of men with localized PCa. For instance, 10‐year all‐cause mortality was estimated to be 20% for curatively treated men diagnosed with intermediate‐risk disease at age 70 years and with a CCI score of 0, compared with 25% for corresponding men in the total group. The 10‐year PCa mortality, however, was slightly different in the curatively treated group. For instance, for men with intermediate‐risk disease and a CCI score of 0 this was estimated to be 4, 5 and 7% diagnosed at age 50, 60 and 70 years in the curatively treated group compared with 2, 4 and 8% in the total group. In relative terms, the proportion of PCa death was higher in those with a CCI score of 0 than those with a CCI score of 2+ (Table S1). The distribution of all other causes of death in curatively treated men was otherwise quite similar to that observed in the total group.

## Discussion

In this nationwide, population‐based study, men with low‐risk PCa had a lower 10‐year all‐cause mortality compared with PCa‐free control subjects, irrespective of comorbidity levels, which was mainly attributable to a lower cardiovascular mortality. The 10‐year PCa mortality for men with low‐risk disease varied between 0.3 and 4%, and PCa was the third most common cause of death among these men, after CVD and other cancers. By contrast, men with intermediate‐ and high‐risk PCa had higher all‐cause mortality than their comparison cohort, mainly driven by death from PCa, which was the most common cause of death among these men.

The uptake of PSA testing has gradually increased in Sweden since the late 1990s 18. This increase in opportunistic screening has caused a drastic rise in the incidence of PCa, as well as a stage migration at time of diagnosis: a twofold increase in the proportion of low‐risk disease (14–28%) and a twofold decrease in the proportion of metastatic disease (25–11%) between 1998 and 2011 in Sweden 19. In a study that modelled PSA screening based on incidence patterns, 56% of Swedish men were estimated to have undergone at least one PSA test in 2007 20. In a study in the Stockholm area in 2011, a direct assessment showed that, at age 50–59 years, 46% of men had undergone PSA testing during the previous 5 years, 68% at age 60–69 years, and 77% at age 70–79 years 21; however, the intensity of PSA testing is still lower in many parts of Sweden in comparison with the USA, where it is estimated that 75% of men aged ≥50 years have had a PSA test 22.

Outcomes of clinically localized PCa managed without primary curative therapy were also investigated in a study based on the Surveillance, Epidemiology and End Results programme. Men aged 78 years at the time of diagnosis had a PCa‐specific mortality of 8% if they had a well‐differentiated cancer, whereas this was 9% for men with a moderately differentiated cancer and 26% for men with a poorly differentiated cancer 23. The present study showed lower numbers, but focused on all treatment groups combined.

Even in a comparison with a matched group of men with no comorbidities, we showed that men with low‐risk PCa had a lower 10‐year all‐cause mortality, which was most likely driven by the lower CVD mortality caused by a self‐selection of health‐conscious men who chose to undergo PSA testing and who are subsequently diagnosed with PCa.

Apart from an early detection selection, differences in PCa stage distribution can also influence mortality from PCa as well as other diseases. This was illustrated in a recent study from the UK, in which 36% of men with localized PCa died from PCa 24. This figure is substantially higher than in all groups in the present study, except for men diagnosed with high‐risk PCa at age 70 years and with high comorbidity (PCa mortality at 10 years: 47%).

The present results for curatively treated men are in accordance with a study of 18 209 men who underwent radical prostatectomy at a US tertial referral centre 25 in which all‐cause mortality was half of that in the general US population, with particularly low death rates for heart disease, chronic respiratory conditions, and diabetes. The proportion of men with CCI = 0 in the US radical prostatectomy cohort was 86% compared with 75% in our low‐risk group, supporting the hypothesis that men who undergo radical prostatectomy are an even more selected group of healthy men. Mortality from other cancers was higher in men with low‐risk PCa than in the comparison cohort and an even higher proportion of death from other cancers was seen in curatively treated men. We have previously shown that men with PCa who are curatively treated have a lower incidence of other primary cancers than men in the general population 26. Thus, the higher mortality from other cancers seems to be driven by the lower competing risk from CVD mortality and not by an increased risk of cancer.

Because the category of low‐risk PCa now constitutes a large proportion of PCa in the Western world and in particular in the USA, all‐cause mortality is a poor outcome measure if the aim is to study the effects of different interventions on the course of PCa; however, all‐cause mortality is still used as an outcome measure for this purpose in contemporary register‐based PCa studies 27. Additionally, the lower CVD mortality in men with low‐risk PCa, compared with the general population, should be taken into account when designing lifestyle interventions intended as primary CVD prevention for these men, as was recently suggested 28.

Our nationwide population‐based cohort with data from several healthcare registers thus enabled us to assess risk and causes of mortality after a diagnosis of localized PCa with unprecedented precision. PCBaSe includes 98% of all new PCa cases diagnosed in Sweden as compared with the Swedish Cancer Register 6. For these cases and for their controls we have information on tumour characteristics at time of diagnosis, primary treatment, socio‐economic status and comorbidity by record linkage to healthcare registers and demographic databases. Moreover, the validity of the Cause of Death Register has been shown to be very high, in particular for men with PCa 29, 30.

In conclusion, in the present nationwide, population‐based study, men with low‐risk PCa had lower all‐cause mortality than PCa‐free men as a result of lower cardiovascular mortality, probably driven by an early detection selection for PSA testing and subsequent diagnostic procedures. For men with intermediate‐ or high‐risk disease PCa, however, the mortality rate was substantial, irrespective of CCI score, and this was even more pronounced for those diagnosed at age 50 or 60 years.

## Conflict of Interest

None declared.

**Abbreviations**
PCa: prostate cancer
NPCR: Swedish National Prostate Cancer Register
CCI: Charlson comorbidity index
ICD: International Classification of Diseases

## Supporting information




**Fig. S1** Predicted risk of death 10 years after prostate cancer (PCa) diagnosis for men aged 50, 60 and 70 years at date of diagnosis, by PCa risk category and Charlson comorbidity index for the subgroup of men curatively treated.

**Table S1** Distribution of causes of death 10 years after prostate cancer (PCa) diagnosis for men aged 50, 60, and 70 years at date of diagnosis, by PCa risk category and Charlson comorbidity index score for the subgroup of men curatively treated.
