# Supplementary figures and images for: Causes of death in men with localized prostate cancer: a nationwide, population‐based study
Source: BJU Int. 2015 May 15;117(3):507–14. doi: 10.1111/bju.13059 (PMC4832314; doi:10.1111/bju.13059)

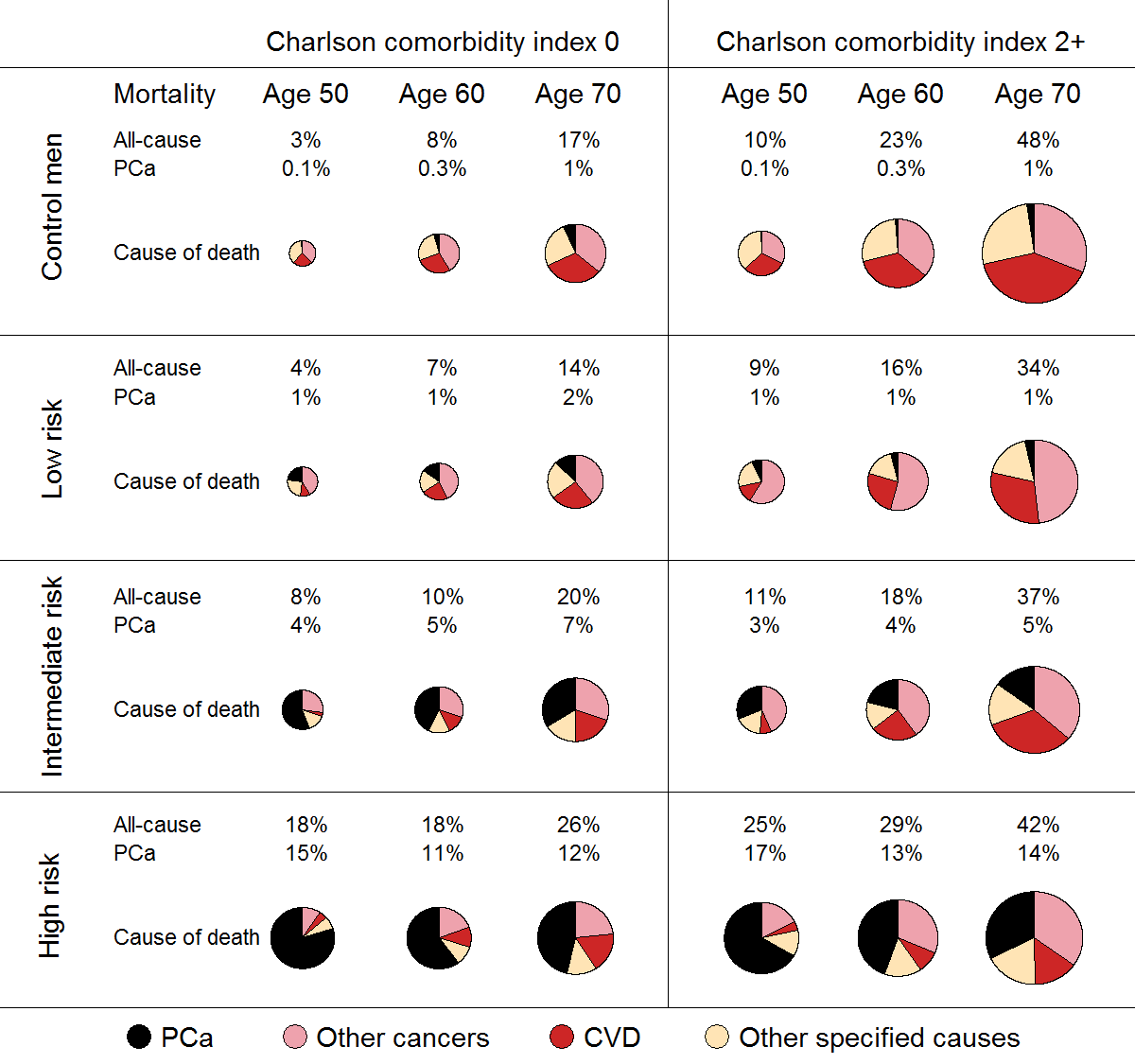

Supplement: Supplementary file 1 — Fig. S1 Predicted risk of death 10 years after prostate cancer (PCa) diagnosis for men aged 50, 60 and 70 years at date of diagnosis, by PCa risk category and Charlson comorbidity index for the subgroup of men curatively treated. [file BJU-117-507-s001.tiff]
