# Supplementary material for: Causes of death in men with localized prostate cancer: a nationwide, population‐based study
Source: BJU Int. 2015 May 15;117(3):507–14. doi: 10.1111/bju.13059 (PMC4832314; doi:10.1111/bju.13059)
Supplement: Supplementary file 2 — Table S1 Distribution of causes of death 10 years after prostate cancer (PCa) diagnosis for men aged 50, 60, and 70 years at date of diagnosis, by PCa risk category and Charlson comorbidity index score for the subgroup of men curatively treated. [file BJU-117-507-s002.docx]

**Table S1** Distribution of causes of death 10 years after PCa diagnosis (for men aged 50, 60, and 70 years at date of diagnosis, by PCa risk category and CCI for the subgroup of men curatively treated. The predictions are made based on median household status (married or in civil partnership) and intermediate educational level. Overall mortality for each category is also shown.

|  | **Charlson comorbidity 0** | | | **Charlson comorbidity 2+** | | |
| --- | --- | --- | --- | --- | --- | --- |
| **Cause of death** | **Age 50** | **Age 60** | **Age 70** | **Age 50** | **Age 60** | **Age 70** |
|  | **Control men** | |  |  |  |  |
| PCa | 2.1 | 4.0 | 6.5 | 0.7 | 1.4 | 2.3 |
| Other cancers | 37.8 | 41.7 | 35.6 | 32.5 | 36.1 | 31.1 |
| CVD | 23.9 | 27.7 | 32.5 | 30.2 | 35.0 | 40.4 |
| Other specified causes | 36.2 | 26.7 | 25.3 | 36.6 | 27.5 | 26.2 |
| Overall mortality | 3.1 | 7.6 | 16.5 | 9.7 | 22.9 | 48.4 |
|  | **Low-risk PCa** | |  |  |  |  |
| PCa | 22.7 | 15.0 | 13.0 | 6.9 | 4.2 | 3.7 |
| Other cancers | 42.5 | 43.1 | 39.2 | 58.5 | 54.0 | 48.3 |
| CVD | 10.1 | 21.7 | 25.6 | 13.1 | 25.6 | 30.1 |
| Other specified causes | 24.7 | 20.2 | 22.3 | 21.5 | 16.2 | 17.9 |
| Overall mortality | 4.1 | 6.6 | 13.9 | 9.4 | 16.3 | 33.9 |
|  | **Intermediate-risk PCa** | | |  |  |  |
| PCa | 55.4 | 43.2 | 34.1 | 31.3 | 20.7 | 15.0 |
| Other cancers | 27.4 | 30.1 | 30.4 | 43.5 | 40.3 | 36.5 |
| CVD | 3.3 | 12.6 | 19.7 | 7.3 | 23.7 | 33.0 |
| Other specified causes | 14.0 | 14.1 | 15.8 | 17.9 | 15.2 | 15.6 |
| Overall mortality | 7.5 | 10.4 | 19.6 | 10.9 | 17.8 | 36.7 |
|  | **High-risk PCa** | |  |  |  |  |
| PCa | 80.0 | 60.3 | 46.4 | 67.0 | 44.5 | 32.4 |
| Other cancers | 9.3 | 19.1 | 23.0 | 17.5 | 31.1 | 34.8 |
| CVD | 3.9 | 10.6 | 17.9 | 3.9 | 9.3 | 14.9 |
| Other specified causes | 6.7 | 9.9 | 12.6 | 11.6 | 15.0 | 18.0 |
| Overall mortality | 18.2 | 18.5 | 25.7 | 24.7 | 28.6 | 41.8 |
